# Academic information on Twitter: A user survey

**DOI:** 10.1371/journal.pone.0197265

**Published:** 2018-05-17

**Authors:** Ehsan Mohammadi, Mike Thelwall, Mary Kwasny, Kristi L. Holmes

**Affiliations:** 1 School of Library and Information Science, University of South Carolina, Columbia, South Carolina, United States of America; 2 Department of Preventive Medicine, Northwestern University Feinberg School of Medicine, Chicago, Illinois, United States of America; 3 Statistical Cybermetrics Research Group, School of Mathematics and Computer Science, University of Wolverhampton, Wolverhampton, United Kingdom; Institut Català de Paleoecologia Humana i Evolució Social (IPHES), SPAIN

## Abstract

Although counts of tweets citing academic papers are used as an informal indicator of interest, little is known about who tweets academic papers and who uses Twitter to find scholarly information. Without knowing this, it is difficult to draw useful conclusions from a publication being frequently tweeted. This study surveyed 1,912 users that have tweeted journal articles to ask about their scholarly-related Twitter uses. Almost half of the respondents (45%) did not work in academia, despite the sample probably being biased towards academics. Twitter was used most by people with a social science or humanities background. People tend to leverage social ties on Twitter to find information rather than searching for relevant tweets. Twitter is used in academia to acquire and share real-time information and to develop connections with others. Motivations for using Twitter vary by discipline, occupation, and employment sector, but not much by gender. These factors also influence the sharing of different types of academic information. This study provides evidence that Twitter plays a significant role in the discovery of scholarly information and cross-disciplinary knowledge spreading. Most importantly, the large numbers of non-academic users support the claims of those using tweet counts as evidence for the non-academic impacts of scholarly research.

## Introduction

Scholars use a range of different types of social web platforms in their professional activities. Twitter is one of the most popular microblogging platforms in many countries, allowing users to broadcast short messages. Academics use Twitter to communicate scientific messages at real-time events, like conferences, but also for more routine information sharing [1]. Since Twitter is used for academic purposes and counts of tweets from Altmetric.com and elsewhere seem to be increasingly consulted for research monitoring [2],[3],[4], it is important to understand the typical motivations for using Twitter in scholarly communication. In response, previous studies have surveyed a small snowball sample of 28 Twitter-active academics [5] and 613 academics with a Twitter account in Physics, Biology, Chemistry, Computer Science, Philosophy, English, Sociology, and Anthropology at 68 U.S. universities [6]. Since tweet counts may be useful as evidence of the impact of research outside of academia (i.e., the extent to which non-academics find a study to be useful), a broader perspective is needed, including non-academic users. In response, this paper reports the first survey of a large and diverse sample of Twitter users inside and outside academia and from around the world to discover who tweets academic publications and why. This contextual information is important to help interpret the meaning of tweet counts as a scholarly impact indicator. Users were selected on the basis that they had tweeted at least one journal article.

The main previous evidence for the value of Tweet counts as impact indicators for academic papers is that they have statistically significant, but small, positive correlations with citation counts in various disciplines, including the medical sciences [7], papers published in arXiv [8], and ecology [9]. These positive associations suggest that the number of times a publication has been shared on Twitter relates in some way to its scholarly impact, but it is difficult to interpret tweet counts confidently because of the low correlations and the possibility that tweets might reflect non-academic impacts. There have also been small scale studies of academic Twitter users, reviewed below, but no investigations of non-academic users. The survey in this article will help to clarify why anyone tweets academic research so that tweet counts can be interpreted with more confidence for the type of impact that they reflect, if any.

The search behavior of Twitter users has studied before but only from a general perspective [10],[11] and little is known about how users find scholarly information. It is therefore not clear whether tweeting academic articles is useful in the sense of helping others to find them.

## Literature review

Scholarly communication in the digital age includes formal and informal uses of the internet to discuss, publish and disseminate scientific discoveries [12]. In the current paper this definition is expanded to cover non-academics accessing, discussing, and sharing academic information. This study focuses on Twitter as a new channel for dissemination of scholarly articles because it is the most widely used general social media site for publication sharing [13]. This section first discusses general reasons for using Twitter in academic contexts. There is no relevant research on academic tweeting (i.e. tweeting scientific articles) by non-academic users, so this issue is not covered. It then focuses on counts of tweets about academic publications as an indicator for research evaluations.

### Types of people using Twitter in a scholarly context

Twitter seems to be used by a substantial minority of academics, although this varies by country. Nothing is known about the extent to which non-academics use Twitter to share scholarly information. About a third (35%) of higher education professionals in the USA were Twitter users in 2010 [14]. Uptake can vary by discipline, with Twitter being among the most popular platforms in the bibliometric community [15]. Internationally, Twitter is banned in some countries and can be second choice to other microblogging services, such as Sina Weibo in China.

### Information discovery on Twitter in academia

Few studies have investigated how people discover scholarly or other information on Twitter. A user study with 20 faculty and students found that facts and links helped to make a tweet useful [16]. A survey of Microsoft employees showed that they sometimes tweeted questions to get factual knowledge, often related to technology [17]. In addition to asking questions and noticing tweets in their feed, users can also search Twitter for a specific piece of information [10]. When users search Twitter, they are typically looking for time-dependent recent information, such as breaking news [11]. In the context of scholarly information, this news might be the publication of new relevant articles or information about an ongoing conference talk, for example. Nevertheless, very little is known about how Twitter is used to actively seek scholarly information.

### Motivations for tweeting in a scholarly context

There are many reasons that academics cite research on Twitter. A survey of 28 tweeting academics in 2010 found that they cited peer-reviewed publications as an integral part of their activities on Twitter [5].

Scholars also use Twitter for purposes other than sharing articles. An online survey of faculty and students of a UK university found that academics use Twitter to increase their professional reputation [18]. An analysis of 68,232 tweets from 37 astrophysicists revealed that they used Twitter to communicate with co-workers, science communicators, other scholars, and related organizations or associations [19]. A study of ten academic disciplines on Twitter found substantial disciplinary differences in usage patterns. For example, cognitive science and digital humanities scholars hosted discussions while economists were more likely to share web links[20]. In contrast, digital humanities scholars use Twitter to get updated information, develop their professional skills, and expand their networks [1]. Most recently, a survey of 515 Twitter users who identified themselves as scientists found that they mainly used Twitter to communicate with researchers and share their publications with the public[21]. An analysis of tweets from 45 academics found that they also share information about their teaching activities and request help or give suggestions[22].

### Types of scholarly content shared on Twitter by academics

At conferences, Twitter is sometimes used to disseminate quotes, notes, and presentations [23]. An analysis of shared links via tweets of three conferences revealed that blog entries, slides shows were among top types of information resources were exchanges [24]. A study of the Social Sciences and Humanities Research Council of Canada (SSHRC) doctoral fellowship recipients showed that they mainly share news links in their tweets even when chatting about scientific subjects [25]. A multidisciplinary analysis of scholars on Twitter indicates that they disseminated informal scientific related resource including magazine papers, blog posts, newspaper links [20].

### Academic topics discussed by non-academics on Twitter

Microblogging services like Twitter may provide a new way for scholars to interact with the public since many non-academics use Twitter and it is suitable for information sharing [26]. Twitter therefore has the potential to supplement or replace existing science communication methods, such as science magazines, public lectures and newspaper stories. Nevertheless, since Twitter is partly a connection-based network, it is not clear whether it can be effective for communication between researchers and non-academics because they may not be part of the same networks. Moreover, the lay public may not search Twitter for scholarly information but may instead consult Wikipedia or the mass media.

There have been systematic attempts by scholars to communicate with the public, indicating a belief that this is worthwhile and possible. For example, one study demonstrated that scholars used Twitter to educate the public in the Flint water crisis in Michigan [27].

A content analysis of 72,469 tweets related to more than 900 scientific news stories revealed that climate change generated the most discussion and there was a complex Twitter ecosystem of contributors that included mass media, celebrity activists, and politicians. Whilst individual scholars and articles did not seem to be important on Twitter for discussions of these news stories, major scientific organizations like @NASA, scientific projects like @marsCuriosity, and prominent scholarly bloggers like literary and cultural commentator Maria Popova (@brainpicker) were important [28]. Thus, it seems possible that typical researchers and papers have little or no impact on the public through Twitter but individual prominent organizations and individuals can be heard. They presumably achieve this through a long-term high-effort strategy of building Twitter networks through quality appropriate content. This would not be practical for most scholars.

Twitter and other social media have been successfully used to engage the public as part of The European Space Agency's comet-chasing Rosetta mission. For example, the hashtag #WakeUpRosetta trended on Twitter at one stage [29]. This illustrates that individual important news stories can engage public attention, and suggests that Twitter might be useful for real-time scientific event monitoring and as part of a wider social and mass media engagement strategy.

From the perspective of journals in a given field, an analysis of academic articles from several conservation studies journals demonstrated that mass media news stories were the most important factor for informing the public about an article, leading to increased sharing on Twitter and Facebook [30]. This confirms that Twitter alone may not be enough to communicate effectively with the public for typical research.

### Scholars liking, saving, and retweeting scholarly content

Liking, saving, and retweeting are important parts of the Twitter information ecosystem. Whilst liking and retweeting help to promote tweets so that they are more easily found by other users, saving helps a user to retrieve a tweet later.

Retweeting can be used to help disseminate academic information. A study of tweets from 447 active scholars from ten disciplines showed that scholars retweeted more (20%-42% of all tweets) than typical users and that biochemists were the most active retweeters [20]. In support of this, Social Sciences and Humanities Research Council doctoral awardees retweeted in 37% of their tweets, although a lower figure (13%) was found for 37 selected Twitter astrophysicists [19]. A study of academic conference tweeters and retweeters found that they had common interests [31] rather than being separate classes of users. Participants at three conferences tended to retweet users with similar opinions and professions [24].

Some projects have studied motivations for favoriting tweets (replaced in 2015 by liking tweets), although not in a scholarly context. A mainly crowdsourced (Tellwut) survey of 606 tweeters in 2013/4 found that half favorited tweets. Users favorited a tweet to help them to find it later or to show approval of the tweet [32] [33]. There seems to be no research focusing on reasons for favoriting academic tweets.

### Tweeting academic publications and research impact

Information about scholarly uses of Twitter is helpful to interpret tweet counts as impact evidence for tweeted publications. Traditionally, citation-based indicators have been used to help evaluate scholarly outputs, but are slow to accumulate and are unable to reflect non-academic impacts [34] [35]. In response, altmetrics have been developed as academic-related indicators derived from social web data, such as Twitter, Mendeley, and blogs, to give earlier evidence of impact or evidence of non-academic impacts [36].

Many publications are mentioned on Twitter. At least 39% of papers submitted to arXiv.org in 2012 have been tweeted at least once [37] and 10% of 1.4 million publications indexed in both PubMed and Web of Science between 2010 and 2012 have been tweeted[38]. More articles are tweeted than mentioned on any other social media platform, according to Altmetric.com [7], although their data may underestimate the numbers of Mendeley readers because this company collects Mendeley information only for articles that are mentioned in at least one other source.

The logical first step for evaluating an impact indicator, such as tweet counts, is to assess whether it correlates with citation counts [39]. This is more straightforward than questionnaires and can give large scale evidence of the relationship between the new indicator and citation counts, which are better understood. Most Twitter studies have found very weak correlations, however. Using Altmetric.com data, an early study found a very low negative correlation between tweets and citation counts [7]. The reason posited for the negative correlation was the rapid increase in Twitter uptake at the time so that new, uncited aritcles were tweeted more than older, cited articles from the same year. A study of publications indexed in both PubMed and Web of Science between 2010 and 2012 found low positive correlations between citations and tweets for articles, with disciplinary variations[38]. For arXiv.org repository papers 2011–2012, downloads and early citation counts correlate moderately with Twitter mentions for academic papers [8]. Weak or moderate correlations between Twitter mentions and citations were found in another study [40], and strong correlations for twenty ecology journals [9]. Using a different approach, tweets of academic articles have been shown to predict future citations, although only for one online journal in the early years of Twitter [41]. Despite some stronger findings, the overall picture is therefore of a very weak relationship between tweet counts and citation counts. This low correlation suggests that people do not tweet articles for the same reason that they cite them. It it is important to investigate these new reasons thoroughly.

Another way to investigate the value of tweeting academic research is to apply content analysis to tweets. Using this approach, most tweets reflect the title of the papers or brief summaries, with very little scholarly discussion [42]. This gives little insight into why an article was tweeted, but rules out critical analyses as a major factor, despite it occurring occasionally [43].

## Research questions

This study investigates key aspects of academic information sharing on Twitter to better understand (a) the context in which academic research is tweeted, and (b) the context in which Twitter is used to find academic information. The research questions cover gaps identified by the literature review.

1. What types of people (occupation; broad disciplinary area; age; gender) tweet academic research?
2. How is Twitter used to find academic-related information?
3. Why is Twitter used to communicate scholarly information? Does the answer depend on academic discipline, gender, age, and occupation?
4. What types of scholarly content is shared on Twitter? Does the answer depend on academic discipline, gender, age, and occupation?
5. Why are scholarly tweets liked, saved, or retweeted?

## Methodology

Although many previous studies have analyzed published tweets, not all tweets originate from a person. Twitter bots create automatic tweets that are difficult to distinguish from human-authored posts [44]. Machines create a substantial number of tweets of academic papers [45], undermining evidence about the value of Twitter from studies that interpret tweets at face value. A survey approach was used to bypass this problem by focusing on human users and to get richer background information.

Twitter had 310 million users in 2017 [46], making it difficult to randomly sample academic-related content and users [23]. In theory, all public tweets could be purchased and data mined to extract a large set of users that generate academic-related tweets. This method is prohibitively costly but a practical alternative would be to use the free Twitter API to randomly sample 1% of all tweets for a specified period and data mine these for academic content. This approach would be undesirable because Twitter does not publicize the methods used to select the permitted 1% of free tweets. Moreover, any data mining attempt to identify academic-related tweeters would introduce its own hidden biases. In response, a novel approach was developed to identify users who shared scientific information through Twitter. A list of Twitter accounts that have tweeted at least one academic paper between January 2011 and December 2015 was obtained from the altmetric data provider Altmetric.com from their ongoing Twitter monitoring of tweets that link to academic domains (e.g., publisher websites) using the commercial Twitter PowerTrack API, giving 4.5 million unique Twitter accounts. Whilst the requirement to have tweeted an academic article is a biasing feature, it is a transparent selection criterion and matches the goal of understanding how academic publication sharing occurs on Twitter.

Twitter’s anti-spam filters made it impossible to tweet a large sample of academic tweeting users directly and so an email survey was used instead. To obtain users’ email addresses, the sample was restricted to users with web links in their Twitter profiles. This is likely to bias the sample towards academic users because these seem most likely to have a public page containing contact information. Such pages are automatically generated for academics by many universities, for example. This additional source of unknown bias is undesirable but there does not seem to be a better alternative. The data mining approach rejected above would also need this step.

Using the data from Altmetric.com, 1,771,520 web links were harvested from the 4.5 million Twitter accounts (i.e., 39%). These links included personal or professional web pages as well as irrelevant targets, such as YouTube videos. The 1,771,520 websites were automatically downloaded and emails extracted from them, when present, producing 57,125 email addresses (1.3% of the original sample) from a range of different domains (Table 1). There were very few emails from all other domains (e.g., .net, .org, .de), and so these were excluded to focus on the main domains. All contact information obtained from the web and Twitter for this study is public data. The data collection methods met terms of service for the websites.

**Table 1:** Email invitations sent to Twitter users and response rates from survey monkey and the Northwestern University mail server.

Domain	Email invitations	Bounced	Opted out	Responses
**.edu**	4508	363	70	460 (11.0%)
**.ac.uk**	2549	671	30	164 (8.7%)
**.ca**	2075	208	26	118 (6.3%)
**.com**	24992	2416	401	737 (3.2%)
**.fr**	985	78	9	26 (2.8%)
**.it**	696	67	2	18 (2.8%)
**.co.uk**	4129	397	52	75 (2.0%)
**Gmail**	16418	388	102	303 (1.8%)
**.nl**	773	74	15	11 (1.5%)
**Total**	57,125	4,662	707	1,912 (3.6%)

A questionnaire was developed and refined through numerous pilot tests. This survey received institutional review board (IRB) approval from Northwestern University. A copy of survey questionnaire is available at https://figshare.com/s/caaa37d468b7302cb611. The questionnaire was sent to 57,125 Twitter users through Survey Monkey in early July 2016, with a reminder sent to non-responding users in late July. Approximately 94% of the invitations were not opened and 1617 emails bounced, giving a low response rate (2%, n = 1105). Some survey invitation emails may have been marked as spam and not received by the intended recipients. Several users emailed to confirm the genuineness of the survey, suggesting that some recipients were reluctant to open the survey link in case it connected to a malicious site.

To increase the response rate, in mid-August 2016, the survey was distributed through Northwestern University, as a trusted academic mail server, to the 53,809 Twitter users who had not opened the Survey Monkey link. Users who had initially opted out of the survey or with bounced email addresses were excluded. A reminder was sent to non-responding invitations in early September. From this, 3,045 additional emails bounced and 113 additional users opted out. This step gave 807 additional responses.

Overall, 1,912 (4%) users completed the survey, with 4,662 (8%) email invitations bouncing and 707 (1%) of users opting out (Table 1). The response rates for academic email addresses (.edu: 11%, .ac.uk: 9%) were higher than for the other domains. The email addresses in commercial domains (Gmail, .com) may not have been the primary or active email accounts of the Twitter users, causing the lower response rates. Alternatively, non-academic users and non-English speakers may have been more reluctant to reply. Most tweets are posted by 20% of all Twitter accounts [47], so the majority of people surveyed may have been occasional tweeters or former users that did not want to bother with a questionnaire about something that was not important to them. The overall response rate, while low, is normal for email questionnaires [48] and web surveys with response rates regularly being lower than 10% [49]. The total number of responses is higher than in other similar surveys with 28 responses [5] and 613 participants [6].

Descriptive statistics are presented as counts and percentages. Chi-square tests were used to assess associations between categorical covariates and responses, Cochran-Armitage trend tests between ordinal covariates and responses, and Wilcoxon rank sum tests for comparisons between skewed continuous covariates and responses. All analyses were performed in SAS v. 9.4, and a nominal 5% type I error level was used.

## Results

The main results are given in the appendices and this section summarizes the key findings.

### Who tweets scholarly information?

Based on the IP addresses of the participants, the survey respondents were 64% from North America, 17% from the United Kingdom, 10% from Europe, 5% from Asia, 2% from South America, 2% from Oceania, and 1% from Africa. This agrees with the overall geographical distribution of Twitter users [50]. There was a slight gender bias in favor of males (55%). The most common age range was 31–40 years old (31%), while 25% were 41–50, 19% were 21–30, 16% were 51–60, 8% were older than 60, and 1% were under 21. Survey participants tend to be younger and male, which is consistent with previous studies [51],[52],[6].

**Fig 1:**
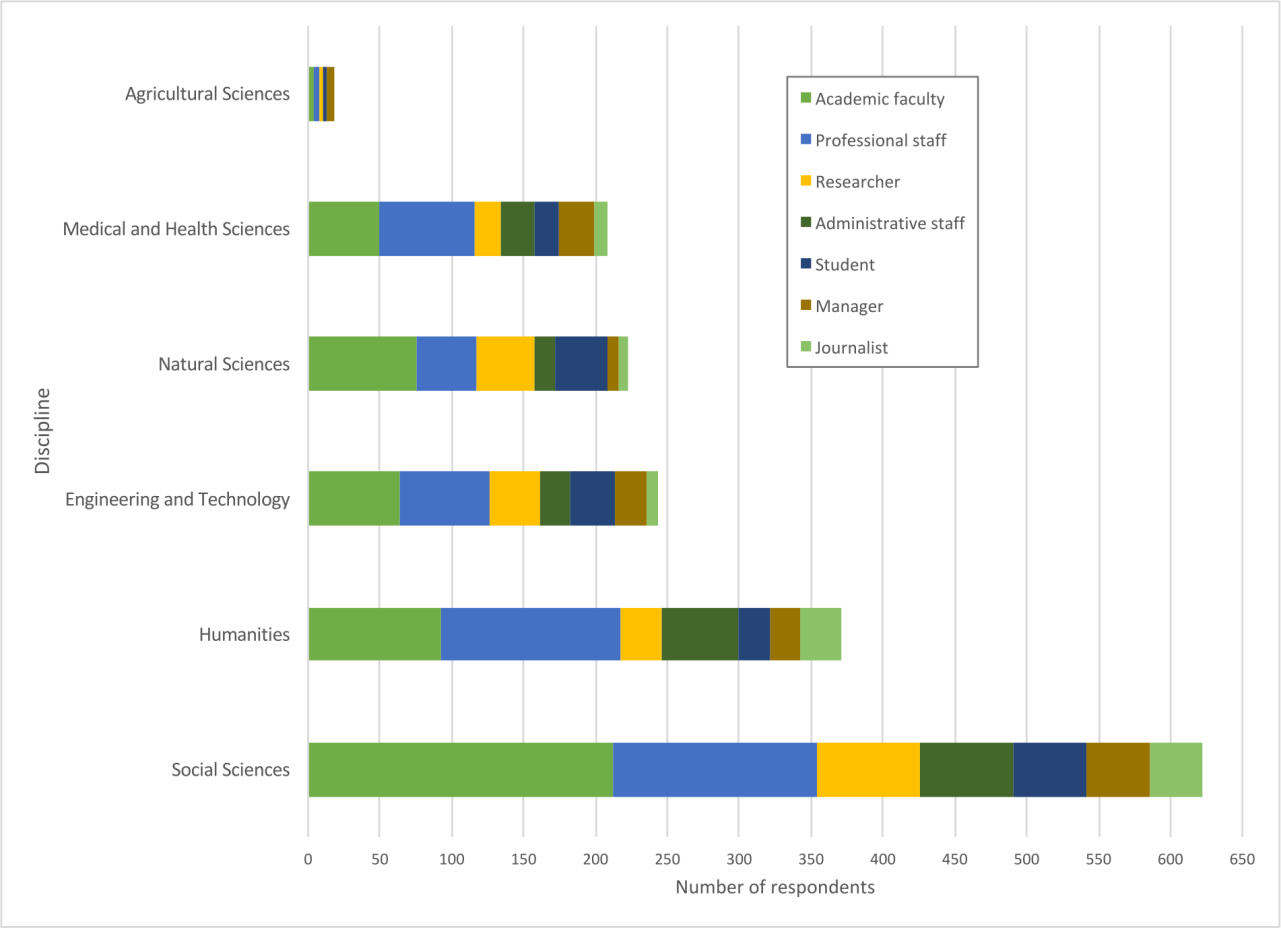
Occupations and academic disciplines declared by the survey participants.

Most participants defined their disciplines as social science (37%) or humanities (22%), with smaller numbers from Engineering/Technology (15%), Natural Sciences (13%), Medical/Health Sciences (11%), and Agricultural Sciences (1%). For the non-academic respondents, these disciplines presumably associated with their highest qualification or occupation. People from these broad disciplinary areas presumably share an information culture to some extent, irrespective of whether they are academics or not. From across academia overall, the first two categories are overrepresented. The survey respondents mostly worked in academic institutions (55%), but almost half worked outside, in industry and professional organizations (41%) or government (4%). Thus, the survey has successfully recruited large numbers of non-academic participants (see also Fig 1).

The academic faculty members were assistant professor/lecturer or senior lecturer (40%), associate professor/reader (25%), professor (25%), instructor/teaching assistant (8%), and adjunct faculty (3%).

### Finding academic-related tweets

Tweets can be found from a user’s Twitter home page, with the Twitter search box and or through general search engines, such as Google and Bing. Most of the respondents (73%, n = 1218 out of 1658 that answered the question) stated that they found tweets by following specific Twitter accounts. Half (50%, n = 826) used hashtags and some used keyword searching through Twitter (43%, n = 718) or web search engines (20%, n = 332). A few used third-party applications, such as TweetDeck (11%, n = 186), and other feeds (5%, n = 82) to find scholarly tweets.

Almost three quarters (73%) of survey participants found academic tweets by following accounts, with insufficient evidence to claim (population) differences between academic disciplines (*p* = .205), and genders (*p* = .205). Account following was more likely to be done by researchers (78%, p<0.001), people in academia (79%, p<0.001), faculty (79%, p<0.001), and younger people (76–77% for 21–40, p = .013). Journalists (62%, p = .001) and older people (for 60+: 64%, p = .013) were least likely to use it. Thus, following accounts is particularly important in an academic context although it is common for all (see S1 Table).

Half of all respondents (50%) searched by hashtags, with insufficient evidence to conclude that there are disciplinary (p = .633), researcher status (p = .814), or work status (p = .831) differences. Hashtags are more often used in academia (53%, p = .024), by females (56%, p<0.001), by younger people (21–30: 58%, p<0.001), and by people that had joined Twitter a longer time ago (8+ years: 57%, p<0.001).

The main disciplinary differences for methods to find tweets concern querying in Twitter or commercial search engines. Humanities scholars used keyword searches in Twitter (48%) much more than did natural scientists (32%). People involved in engineering or technology used commercial search engines (25%) more than did natural scientists (17%). The underlying difference here is that natural scientists are less likely to actively search for tweets than are users from other disciplines.

Non-researchers are more likely to search in Twitter (46% vs. 43%) or commercial search engines (22% vs. 18%) than are researchers, but they otherwise find tweets in similar ways.

There are differences between sectors in all methods used to find tweets, except for 3^rd^ party apps. People in academia are the most likely to follow accounts (79%, compared to 70% for government, 67% for commercial/professional, p<0.001) and hashtags (53%, 48%, 46%, p = .024). Government users are most likely to use a commercial search engine (15%, 28%, 26%, p<0.001), whereas professional/commercial users are most likely to use Twitter keyword searches (40%, 36%, 48%, p = .002). Thus, more active searching is more characteristic of non-academic users.

The only statistically significant difference in methods to find tweets is that females are more likely to follow hashtags (56% vs. 45%, p<0.001). Hashtags probably perform a communicative function often in academia, such as by allowing conference participants to interact online.

There are age differences in all methods used to find tweets, except for 3^rd^ party apps. The biggest difference is that older users (60+: 31%) were almost twice as likely to find tweets with a commercial search engine than were younger users (<21: 16%).

### Reasons for using Twitter in academic settings

The survey respondents that gave a reason for using Twitter (n = 1811) used it to obtain real-time information (73%, n = 1323), share real-time information (66%, n = 1198), expand their professional networks (64%, n = 1150), contribute to wider conversations (54%, n = 985) promote organizations (55%, n = 995), communicate about academic events (52%, n = 949), communicate the results of scientific publications to the public (47%, n = 856) and peers (43%, n = 773), and for teaching (16%, n = 292) (see S2 Table).

There were no statistically significant differences between disciplines in terms of the extent to which respondents obtained (*p* = .535) or shared (*p* = .390) real-time information, or used Twitter to expand their professional network (*p* = .650). In Social Sciences, Twitter is more likely to be used in teaching. In Humanities, it is more likely to be used to contribute to wider conversations, a natural humanities role. In Engineering/Technology, Twitter tends to be used less for everything, particularly for communicating results and about academic events. In Natural Sciences, it is less likely to be used to contribute to wider conversations. In Medical/Health Sciences, Twitter tends to be used more for most things except teaching, and is particularly well used for communicating results to peers and the public and for communicating about academic events (see S2 Table).

Unsurprisingly, researchers are more likely to communicate research results but non-researchers are more likely to tweet promoting organizations. Similarly, in terms of work sector, the use of Twitter is higher in academia for most things except promoting organizations, where is it more common in industry. Government workers are the most likely to Tweet to contribute to wider conversations. Nevertheless, journalists use Twitter more for finding and sharing real-time information and for contributing to wider conversations, which fits their job role. Managers use it the most for promoting their organization (see S2 Table).

More males used Twitter to promote their organizations and communicate about academic events than did females (*p =* .001). There were no other significant gender differences in the other reasons for using Twitter in scholarly contexts (see S2 Table).

Age was a statistically significant factor for obtaining real-time information (*p =* .001), communicating the results of academic publications to peers (*p =* .001) and the public (*p =* .001), communicating about academic events (*p =* .001), teaching (*p =* .003) and promoting their organization (*p =* .001). In general, people aged 31–40 tended to have more reasons for using Twitter and people under 21 tended to have the fewest reasons (see S2 Table).

### What types of scholarly content are shared through Twitter?

Survey participants shared many different types of academic-related materials on Twitter, including research articles and other published works (77%, n = 1281), research related news (68%, n = 1135), blog posts (66%, n = 1097), lay summaries of research for the public (42%, n = 703), videos and images (33%, n = 548), policy announcements (31%, n = 508), and presentation slides (20%, n = 338) (S3 Table). Since participants were selected on the basis that they had tweeted an academic article, the 77% figure above suggests that many users (23%) had forgotten some of their tweets.

Social scientists tended to share the most, including publications, research-related news and policy announcements. Humanities scholars tended to share the least, including publications, slides, and research-related news. In Engineering/Technology, slides were shared the most, presumably reflecting the importance of conferences in many engineering fields. In Medical/Health Sciences, policy announcements were frequently shared (S3 Table).

Unsurprisingly, researchers shared the most publications and research-related news. Interestingly, however, non-researchers shared the most videos and images, blog posts, and lay summaries. Thus, there seems to be a niche role for non-researchers in helping to communicate research in non-standard ways. This is broadly echoed by the work sector results. The government and industry/professional sectors tend to share similarly, except that government workers share more research-related news. In terms of occupation, researchers and faculty tended to share publications, presentations, and research-related news the most and lay summaries, videos, and images the least (S3 Table).

There were only minor gender differences in sharing, with males sending to share presentations more (perhaps due to their use in male-dominated engineering fields), whereas females shared research-related news and lay summaries more. The age range 31–50 was the most active for sharing, including publications, research-related news. People with older Twitter accounts also tended to share more, including presentations, videos and images, and blog posts (S3 Table).

### The role of Twitter in scholarly practice

**Fig 2:**
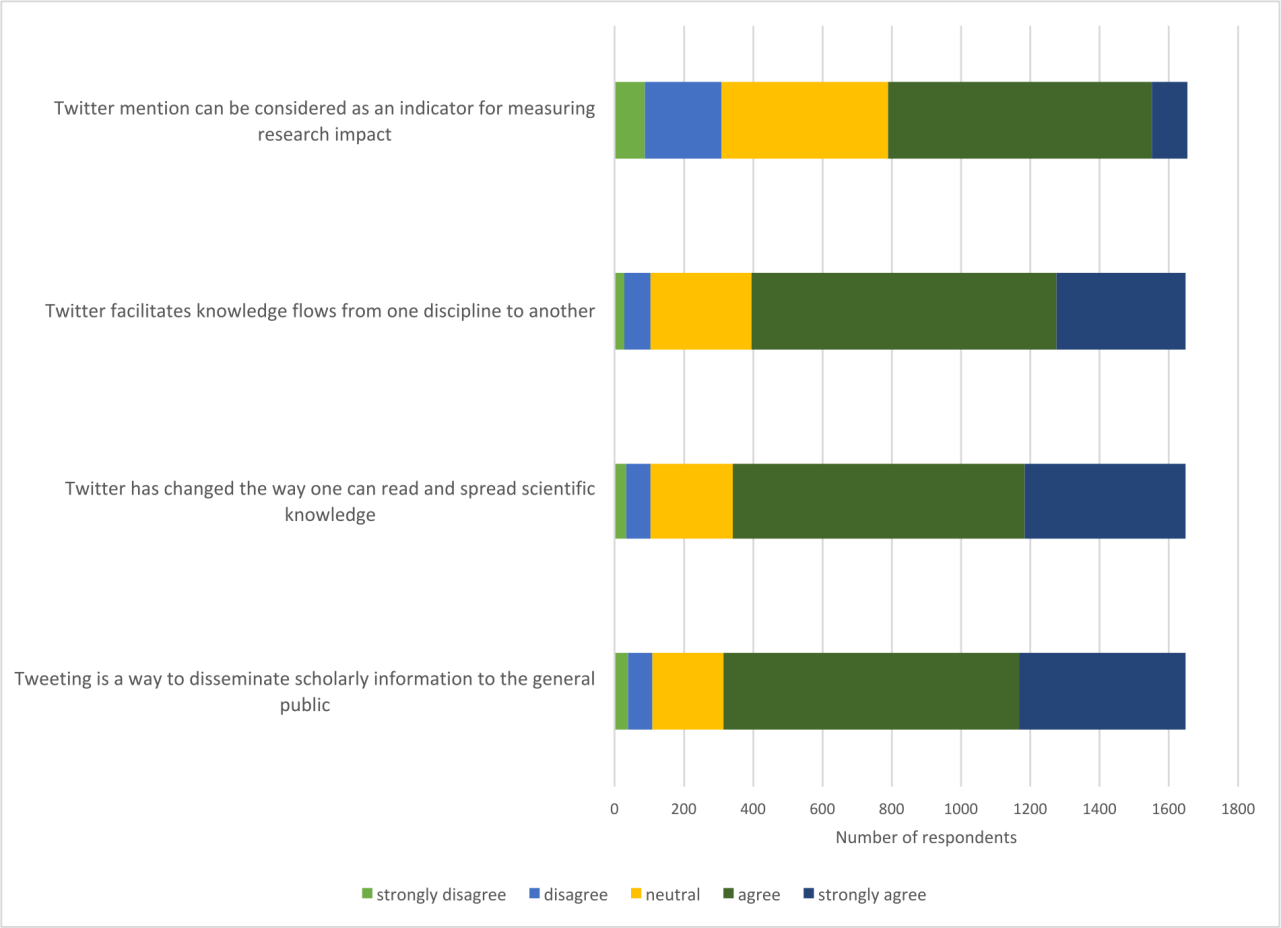
The role of Twitter in online scholarly communication.

Several questions were asked about the use of Twitter in scholarly activities. Most respondents agreed or strongly agreed (81%, n = 1333) that tweeting academic articles disseminates scholarly information to the public, representing a broad consensus (Fig 2), despite a lack of evidence that the public read academic tweets in most fields (health and astronomy are exceptions). Most respondents also agreed or strongly agreed (79%, n = 1307) that Twitter has changed the way that academic knowledge can be read and disseminated. Almost as many respondents agreed or strongly agreed (76%, n = 1249) that Twitter facilitates knowledge flows from one academic discipline to another.

Although respondents agreed that Twitter could be used to communicate scientific knowledge, they were split about whether it could be used to measure scholarly impact. Only half (52%, n = 864) agreed or strongly agreed, with nearly one-third (29%, n = 479) being neutral.

### User activities and influences in Twitter in scientific contexts

Two thirds of survey respondents (66%) tweeted at least weekly. This was most common in Social Sciences (69%) and least common in Humanities and Engineering/Technology (62%). Journalists were the most likely to tweet at least weekly (80%) and professionals (58%) the least likely. Males (68%) and people aged 51–60 (70%) were more frequent tweeters, as were people with an account over 8 years old (71%) (S4 Table).

Users have several engagement options available on Twitter: *liking* (formerly known as favoriting), *saving*, and *retweeting* content. Most respondents (71%, n = 1197) *like* a tweet to inform the authors that their tweets were interesting. Forty-three percent (n = 725) *like* tweets to help them to be found in the future. Only a fifth (21%, n = 352) *liked* tweets to disseminate them (Table 2).

**Table 2:** Motivations to like, save and retweet on Twitter.

Action	Disseminate	Save or access later	Let author know I read it	Let author know I found it interesting
**Like/favorite**	352 (21.0%)	725 (43.2%)	488 (29.1%)	1197 (71.4%)
**Retweet**	1428 (85.2%)	351 (20.9%)	253 (15.0%)	710 (42.3%)
**Save**	76 (4.5%)	790 (47.1%)	56 (3.3%)	86 (5.1%)

Unsurprisingly, academic tweets were usually *retweeted* to disseminate them (85%, n = 1428) but also sometimes (42%, n = 710) to tell the author that their tweet was interesting, for future access (21%, n = 351) and to inform the author that they had read the tweet (15%, n = 235).

## Limitations

The results have several limitations. The survey sample is limited to Twitter users with personal webpage links in their Twitter profiles. These are presumably more likely to be academics or professionals than people that are unemployed or working in government or industry. The sample is also restricted to people that have current email addresses in their webpages that can be extracted using web-mining methods. Thus, the original sample is a limited and probably biased subset of people that have tweeted academic articles. The low response rate is typical of online surveys and is likely to bias the results to the attitudes of people that have a greater interest in the use of Twitter within research. This may explain the high numbers of social sciences and humanities scholars. It probably also biases the results towards more active users.

The numerous statistical tests greatly increase the likelihood that at least one finds spurious evidence of differences when none are present (i.e., a high familywise type 1 error likelihood). The results should therefore be interpreted cautiously, especially when the *p* value is close to 0.05. As with all surveys, some respondents may misinterpret the questions. Moreover, social network sites and typical methods of use change over time, adding extra uncertainty to the results.

## Discussion

The survey suggests that most people who tweet academic information work in academic institutions, with other groups of users outside academia such professionals (engineers, physicians, and lawyers), but with many managers and journalists also tweeting academic information. This is consistent with previous research [31],[13], but probably over-represents academia due to the survey sampling method (emails from home page URLs in Twitter profiles). In this context, the relatively high proportion of non-academic tweeters is a surprising and important finding. No previous survey of Twitter use for scholarly communication has attempted to identify non-academic tweeters of academic research. For people seeking to use Twitter to disseminate information to the wider public, this is the most substantial evidence yet that this may work.

Within academia, the level of Twitter use varies by academic rank, with assistant professors among the top users, followed by associate professors, corroborating previous research [6]. Most respondents tweeting scholarly information were from the social sciences or humanities, agreeing with previous studies [20],[53],[13].

In terms of gender, the greater number of males responding to the survey may reflect similar gender imbalances in science [54] and perhaps also for Twitter use in academia [55].

Following specific accounts and hashtags are the primary means used to find academic information on Twitter. Users prefer this to searching on Twitter or via a web search engine. This probably reflects the way in which most people use Twitter [56] rather than being specific to science. Thus social ties are important on Twitter for finding information, and the same may also be said for hashtags, since these are human-generated communication channels [10].

For the second research question, the primary motivations for using Twitter in scholarly contexts are to get and share real-time information, and to develop connections. Twitter is also used to participate in online conversations, increase the visibility of organizations, disseminate activities related to academic events, share scholarly findings with peers and the public, and for educational purposes. These findings are consistent with previous studies about the reasons for using Twitter in general [57] and academic purposes in particular [58],[18]. There were statistically significant differences by discipline, employment sector, and occupation for many of these motivating factors.

Scientific publications, academic news, and blog posts are the most common information sources shared on Twitter. Some respondents also share lay summaries of research, videos, images, policy documents, and presentations. These findings are in agreement with an earlier study that used link analysis [13]. There were statistically significant differences by discipline, employment sector, occupation, age, and gender. For instance, faculty shared research articles more than other users while professionals tweeted scholarly videos the most. These differences are broadly consistent with what might be expected.

The consensus (81%) that tweeting academic articles disseminates scholarly information to the public is given some credence by the substantial number of non-academics responding to this survey. Perhaps related to this, most respondents (52%) thought that Twitter mentions of academic publications can be a research impact metric. Given the low correlations between Twitter mentions of scientific articles and citation counts found in previous studies[38],[8],[59], it is possible that tweets reflect non-academic impacts to a wider extent than previously thought. This supports previously unsubstantiated claims that Twitter mentions of academic papers could reflect their societal impact,which is an important issue in research evaluation [60].

There was agreement (81%) that Twitter has changed the way that scientific knowledge can be read and spread, including between disciplines (76%). Although previous studies have found evidence of cross-disciplinary knowledge transfer for other social web sites [61],[62] this is the first large-scale evidence for Twitter.

Most participants composed, replied to, liked and retweeted tweets. The level of activity varied based on the respondents’ occupation and gender, with males more actively tweeting than females. Researchers and faculty members at academic institutions more actively tweeted scholarly information than other users.

## Conclusions

The results have several practical implications. For information sharing, Twitter is particularly important in the social sciences and humanities. Thus, researchers in these areas should be encouraged to investigate whether they can benefit from using the service, if they are not already doing so. It is possible that other disciplines rely on effective alternative communication means, such as conferences, preprint archives, or professional online networks such as LinkedIn.

Since users rely on social connections to find information on Twitter [10], people that seek to disseminate information on Twitter should not rely on others finding it through keyword searches and should try to build up followers or use appropriate hashtags instead. This will be unsurprising to experienced users.

The dissemination of science to wider audiences is an increasingly important task in academia. People that use Twitter to communicate research agree that it can perform this role, and this is supported by the proportion of non-academics responding to the survey (46% chose Government or Industry/Professional rather than Academic as their work sector) that also do this, despite the likely bias of the survey towards academics (because email addresses were extracted from personal pages to find respondents). This gives the strongest evidence yet that Twitter may be successful in this role and that Twitter-based indicators may also help to reveal the wider impacts of scholarly publications–although not in formal evaluations [63].

Finally, since messages shared on Twitter are not always conveyed effectively [31],[42],[64] and the current study has found a substantial number of non-academics that are interested enough in research to tweet papers, it is now increasingly important for academics to be able to write tweets that are understandable by a lay audience.

## Supporting information

S1 Table Methods used to find academic tweets based on academic discipline, age, gender and current job. (DOCX)

S2 Table Primary reasons for scholarly use of Twitter by academic discipline, gender, age and occupation. (DOCX)

S3 Table Types of scientific content shared by users on Twitter by discipline, gender, age and job. (DOCX)

S4 Table The level of scholarly activity on Twitter by discipline, gender, age and occupation. (DOCX)
