# Supplementary material for: Academic information on Twitter: A user survey
Source: PLoS One. 2018 May 17;13(5):e0197265. doi: 10.1371/journal.pone.0197265 (PMC5957360; doi:10.1371/journal.pone.0197265)
Supplement: S1 Table — (DOCX) [file pone.0197265.s001.docx]

**Supplemental Data**

# S1 Table. Methods used to find academic tweets based on academic discipline, age, gender, and current job.

|  | **Following particular accounts (n, %)** | **Hashtags (n, %)** | **Keyword (n, %)** | **Search engine (n, %)** | **3rd party tools (n, %)** | **Only read feeds (n, %)** | **Answered question** |
| --- | --- | --- | --- | --- | --- | --- | --- |
| **Academic Background** | | | | | | |  |
| Social Sciences | 466 (76%) | 323 (53%) | 274 (45%) | 108 (18%) | 66 (11%) | 31 (5%) | 610 |
| Humanities | 264 (73%) | 180 (50%) | 174 (48%) | 74 (20%) | 45 (12%) | 16 (4%) | 363 |
| Engineering/Technology | 165 (69%) | 112 (47%) | 102 (43%) | 59 (25%) | 27 (11%) | 10 (4%) | 239 |
| Natural Sciences | 169 (76%) | 104 (47%) | 70 (32%) | 37 (17%) | 22 (10%) | 12 (5%) | 221 |
| Medical/Health Sciences | 143 (69%) | 99 (48%) | 90 (43%) | 49 (24%) | 24 (12%) | 11 (5%) | 207 |
| Agricultural Sciences | 11 (61%) | 8 (44%) | 8 (44%) | 5 (28%) | 2 (11%) | 2 (11%) | 18 |
| **Total** | **1218 (73%)** | **826 (50%)** | **718 (43%)** | **332 (20%)** | **186 (11%)** | **82 (5%)** | **1658** |
| **p-value** | **0.205** | **0.633** | **0.01** | **0.078** | **0.966** | **0.81** |  |
| **Researcher status** | | | | | | |  |
| Researcher | 728 (78%) | 473 (51%) | 382 (41%) | 169 (18%) | 98 (11%) | 40 (4%) | 933 |
| Not researcher | 490 (68%) | 353 (49%) | 336 (46%) | 163 (22%) | 88 (12%) | 42 (6%) | 725 |
| **Total** | **1218 (73%)** | **826 (50%)** | **718 (43%)** | **332 (20%)** | **186 (11%)** | **82 (5%)** | **1658** |
| **p-value** | **<0.001** | **0.814** | **0.008** | **0.013** | **0.211** | **0.125** |  |
| **Work sector** | | | | | | |  |
| Academia | 730 (79%) | 490 (53%) | 373 (40%) | 141 (15%) | 104 (11%) | 32 (3%) | 929 |
| Government | 45 (70%) | 31 (48%) | 23 (36%) | 18 (28%) | 6 (9%) | 4 (6%) | 64 |
| Industry/Professional | 443 (67%) | 305 (46%) | 322 (48%) | 173 (26%) | 76 (11%) | 46 (7%) | 665 |
| **Total** | 1218 (73%) | 826 (50%) | 718 (43%) | 332 (20%) | 186 (11%) | 82 (5%) | **1658** |
| **p-value** | **<0.001** | **0.024** | **0.002** | **<0.001** | **0.868** | **0.006** |  |
| **Current position** | | | | | | |  |
| Faculty | 388 (79%) | 237 (48%) | 162 (33%) | 56 (11%) | 43 (9%) | 21 (4%) | 493 |
| Professional | 300 (69%) | 214 (50%) | 213 (49%) | 111 (26%) | 44 (10%) | 26 (6%) | 432 |
| Researcher | 154 (79%) | 100 (51%) | 87 (44%) | 34 (17%) | 19 (10%) | 13 (7%) | 196 |
| Administrator | 122 (71%) | 86 (50%) | 72 (42%) | 39 (23%) | 19 (11%) | 4 (2%) | 173 |
| Student | 115 (75%) | 82 (53%) | 83 (54%) | 29 (19%) | 22 (14%) | 4 (3%) | 154 |
| Manager | 83 (69%) | 66 (55%) | 52 (43%) | 33 (27%) | 21 (17%) | 7 (6%) | 121 |
| Journalist | 54 (62%) | 41 (47%) | 48 (55%) | 29 (33%) | 18 (21%) | 7 (8%) | 87 |
| **Total** | **1216 (73%)** | **826 (50%)** | **717 (43%)** | **331 (20%)** | **186 (11%)** | **82 (5%)** | **1656** |
| **p-value** | **0.001** | **0.831** | **<0.001** | **<0.001** | **0.006** | **0.173** | 493 |
| **Gender** | | | | | | |  |
| Males | 669 (74%) | 402 (45%) | 375 (42%) | 188 (21%) | 99 (11%) | 43 (5%) | 903 |
| Females | 540 (72%) | 420 (56%) | 338 (45%) | 143 (19%) | 87 (12%) | 39 (5%) | 745 |
| **Total** | **1209 (73%)** | **822 (50%)** | **713 (43%)** | **331 (20%)** | **186 (11%)** | **82 (5%)** | **1648** |
| **p-value** | **0.437** | **<0.001** | **0.123** | **0.405** | **0.655** | **0.665** |  |
| **Age**** | | | | | | |  |
| <21 | 3 (30%) | 3 (30%) | 6 (60%) | 4 (40%) | 1 (10%) | 1 (10%) | 10 |
| 21-30 | 231 (76%) | 176 (58%) | 142 (47%) | 50 (16%) | 34 (11%) | 10 (3%) | 304 |
| 31-40 | 403 (77%) | 269 (51%) | 235 (45%) | 89 (17%) | 66 (13%) | 16 (3%) | 525 |
| 41-50 | 305 (73%) | 215 (52%) | 173 (42%) | 79 (19%) | 45 (11%) | 22 (5%) | 415 |
| 51-60 | 183 (71%) | 112 (43%) | 107 (41%) | 67 (26%) | 25 (10%) | 17 (7%) | 259 |
| 60+ | 87 (64%) | 47 (34%) | 50 (36%) | 42 (31%) | 14 (10%) | 16 (12%) | 137 |
| **Total** | **1212 (73%)** | **822 (50%)** | **713 (43%)** | **331 (20%)** | **185 (11%)** | **82 (5%)** | **1650** |
| **p-value** | **0.013** | **<0.001** | **0.018** | **<0.001** | **0.389** | **<0.001** |  |
| **Age of Twitter account, years** | | | | | | |  |
| <1 | 18 (69%) | 7 (27%) | 9 (35%) | 3 (12%) | 1 (4%) | 2 (8%) | 26 |
| 1-2 | 102 (72%) | 54 (38%) | 48 (34%) | 28 (20%) | 10 (7%) | 7 (5%) | 142 |
| 2-5 | 497 (72%) | 335 (49%) | 293 (43%) | 134 (19%) | 54 (8%) | 35 (5%) | 688 |
| 5-8 | 477 (76%) | 333 (53%) | 271 (43%) | 121 (19%) | 90 (14%) | 31 (5%) | 628 |
| 8+ | 122 (72%) | 96 (57%) | 95 (56%) | 46 (27%) | 31 (18%) | 6 (4%) | 169 |
| **Total** | **1216 (74%)** | **825 (50%)** | **716 (43%)** | **332 (20%)** | **186 (11%)** | **81 (5%)** | **1653** |
| **p-value** | **0.321** | **<0.001** | **<0.001** | **0.083** | **<0.001** | **0.434** |  |

*P-value from chi-square test unless otherwise noted. **p-value from Kruskal-Wallis test.
