# Supplementary material for: Academic information on Twitter: A user survey
Source: PLoS One. 2018 May 17;13(5):e0197265. doi: 10.1371/journal.pone.0197265 (PMC5957360; doi:10.1371/journal.pone.0197265)
Supplement: S2 Table — (DOCX) [file pone.0197265.s002.docx]

# S2 Table. Primary reasons for scholarly use of Twitter by academic discipline, gender, age, and occupation.

|  | **Obtain real-time information** | **Share real-time information** | **Communicate results to peers** | **Communicate results to public** | **Communicate about academic events** | **Contribute to wider conversations** | **To use in teaching** | **To expand professional network** | **To promote organization** | **Answered question** |
| --- | --- | --- | --- | --- | --- | --- | --- | --- | --- | --- |
| **Academic background** | | | | | | | | | |  |
| Social Sciences | 493 (74%) | 457 (69%) | 311 (47%) | 340 (51%) | 356 (53%) | 369 (55%) | 135 (20%) | 433 (65%) | 372 (56%) | 666 |
| Humanities | 288 (72%) | 264 (66%) | 144 (36%) | 172 (43%) | 220 (55%) | 239 (60%) | 67 (17%) | 254 (64%) | 236 (59%) | 398 |
| Engineering/Technology | 191 (72%) | 168 (63%) | 94 (35%) | 96 (36%) | 115 (43%) | 137 (52%) | 29 (11%) | 173 (65%) | 127 (48%) | 266 |
| Natural Sciences | 183 (76%) | 153 (63%) | 107 (44%) | 116 (48%) | 128 (53%) | 117 (49%) | 37 (15%) | 142 (59%) | 114 (47%) | 241 |
| Medical/Health Sciences | 154 (69%) | 145 (65%) | 109 (49%) | 124 (56%) | 124 (56%) | 114 (51%) | 21 (9%) | 136 (61%) | 138 (62%) | 222 |
| Agricultural Sciences | 14 (78%) | 11 (61%) | 8 (44%) | 8 (44%) | 6 (33%) | 9 (50%) | 3 (17%) | 12 (67%) | 8 (44%) | 18 |
| **Total** | 1323 (73%) | 1198 (66%) | 773 (43%) | 856 (47%) | 949 (52%) | 985 (54%) | 292 (16%) | 1150 (64%) | 995 (55%) | 1811 |
| **p-value** | **0.535** | **0.39** | **0.001** | **<0.001** | **0.01** | **0.02** | **0** | **0.65** | **0** |  |
| **Researcher status** | | | | | | | | | |  |
| Researcher | 740 (75%) | 651 (66%) | 558 (57%) | 559 (57%) | 618 (63%) | 526 (53%) | 190 (19%) | 617 (63%) | 488 (49%) | 987 |
| Not researcher | 583 (71%) | 547 (66%) | 215 (26%) | 298 (36%) | 331 (40%) | 459 (56%) | 102 (12%) | 533 (65%) | 507 (62%) | 824 |
| **Total** | 1323 (73%) | 1198 (66%) | 773 (43%) | 857 (47%) | 949 (52%) | 985 (54%) | 292 (16%) | 1150 (64%) | 995 (55%) | 1811 |
| **p-value** | **0.705** | **0.018** | **<0.001** | **<0.001** | **<0.001** | **0.01** | **0** | **0.39** | **<0.001** |  |
| **Work sector** | | | | | | | | | |  |
| Academia | 727 (74%) | 646 (65%) | 541 (55%) | 542 (55%) | 673 (68%) | 510 (52%) | 214 (22%) | 614 (62%) | 509 (52%) | 987 |
| Government | 54 (79%) | 46 (68%) | 23 (34%) | 29 (43%) | 30 (44%) | 43 (63%) | 10 (15%) | 39 (57%) | 33 (49%) | 68 |
| Industry/Professional | 542 (75%) | 506 (70%) | 209 (29%) | 285 (39%) | 245 (34%) | 432 (60%) | 68 (9%) | 468 (64%) | 453 (62%) | 726 |
| **Total** | 1323 (74%) | 1198 (67%) | 773 (43%) | 856 (48%) | 948 (53%) | 985 (55%) | 292 (16%) | 1121 (63%) | 995 (56%) | 1781 |
| **p-value** | **0.151** | **0.011** | **<0.001** | **<0.001** | **<0.001** | **<0.001** | **<0.001** | **0.44** | **<0.001** |  |
| **Current position** | | | | | | | | | |  |
| Administrator | 121 (63%) | 118 (61%) | 57 (30%) | 73 (38%) | 108 (56%) | 89 (46%) | 17 (9%) | 123 (64%) | 125 (65%) | 193 |
| Faculty | 368 (72%) | 345 (68%) | 307 (60%) | 294 (58%) | 351 (69%) | 289 (57%) | 160 (31%) | 322 (63%) | 264 (52%) | 508 |
| Journalist | 76 (83%) | 70 (76%) | 25 (27%) | 42 (46%) | 31 (34%) | 61 (66%) | 6 (7%) | 59 (64%) | 54 (59%) | 92 |
| Manager | 99 (74%) | 98 (73%) | 42 (31%) | 62 (46%) | 50 (37%) | 81 (60%) | 13 (10%) | 80 (60%) | 96 (72%) | 134 |
| Professional | 354 (75%) | 327 (69%) | 137 (29%) | 182 (38%) | 188 (40%) | 281 (59%) | 61 (13%) | 305 (64%) | 293 (62%) | 473 |
| Researcher | 162 (77%) | 131 (62%) | 128 (61%) | 126 (60%) | 131 (62%) | 103 (49%) | 24 (11%) | 124 (59%) | 100 (48%) | 210 |
| Student | 140 (84%) | 107 (64%) | 75 (45%) | 75 (45%) | 89 (53%) | 80 (48%) | 10 (6%) | 106 (63%) | 62 (37%) | 167 |
| **Total** | 1320 (74%) | 1196 (67%) | 771 (43%) | 854 (48%) | 948 (53%) | 984 (55%) | 291 (16%) | 1119 (63%) | 994 (56%) | 1777 |
| **p-value** | **<0.001** | **0.029** | **<0.001** | **<0.001** | **<0.001** | **0** | **<0.001** | **0.88** | **<0.001** |  |
| **Gender** | | | | | | | | | |  |
| Males | 719 (78%) | 637 (69%) | 421 (45%) | 453 (49%) | 477 (51%) | 534 (58%) | 161 (17%) | 563 (61%) | 504 (54%) | 927 |
| Female | 598 (78%) | 554 (73%) | 345 (45%) | 396 (52%) | 465 (61%) | 445 (58%) | 128 (17%) | 470 (62%) | 483 (63%) | 762 |
| **Total** | 1317 (78%) | 1191 (71%) | 766 (45%) | 849 (50%) | 942 (56%) | 979 (58%) | 289 (17%) | 1033 (61%) | 987 (58%) | 1689 |
| **p-value** | **0.651** | **0.074** | **0.95** | **0.205** | **<0.001** | **0.74** | **0.84** | **0.63** | **<0.001** |  |
| **Age**** | | | | | | | | | |  |
| <21 | 8 (80%) | 7 (70%) | 0 (0%) | 1 (10%) | 2 (20%) | 6 (60%) | 0 (0%) | 6 (60%) | 5 (50%) | 10 |
| 21-30 | 269 (84%) | 221 (69%) | 124 (39%) | 130 (41%) | 166 (52%) | 169 (53%) | 32 (10%) | 196 (61%) | 149 (47%) | 319 |
| 31-40 | 427 (80%) | 369 (69%) | 292 (55%) | 302 (57%) | 342 (64%) | 306 (57%) | 100 (19%) | 331 (62%) | 312 (59%) | 533 |
| 41-50 | 339 (80%) | 304 (72%) | 206 (49%) | 221 (52%) | 237 (56%) | 248 (59%) | 83 (20%) | 255 (60%) | 266 (63%) | 423 |
| 51-60 | 180 (68%) | 188 (71%) | 93 (35%) | 125 (48%) | 143 (54%) | 160 (61%) | 53 (20%) | 149 (57%) | 176 (67%) | 263 |
| 60+ | 95 (66%) | 104 (72%) | 53 (37%) | 70 (49%) | 56 (39%) | 92 (64%) | 22 (15%) | 96 (67%) | 81 (56%) | 144 |
| **Total** | 1318 (78%) | 1193 (71%) | 768 (45%) | 849 (50%) | 946 (56%) | 981 (58%) | 290 (17%) | 1033 (61%) | 989 (58%) | 1692 |
| **p-value** | **<0.001** | **0.904** | **<0.001** | **<0.001** | **<0.001** | **0.24** | **0** | **0.46** | **<0.001** |  |
| **Age of Twitter account, years** | | | | | | | | | |  |
| <1 | 18 (62%) | 16 (55%) | 8 (28%) | 10 (34%) | 17 (59%) | 8 (28%) | 2 (7%) | 17 (59%) | 10 (34%) | 29 |
| 1-2 | 104 (71%) | 80 (55%) | 76 (52%) | 75 (51%) | 87 (60%) | 65 (45%) | 21 (14%) | 89 (61%) | 87 (60%) | 146 |
| 2-5 | 505 (72%) | 473 (68%) | 337 (48%) | 362 (52%) | 395 (57%) | 390 (56%) | 116 (17%) | 429 (61%) | 411 (59%) | 698 |
| 5-8 | 550 (85%) | 490 (76%) | 280 (43%) | 320 (49%) | 347 (53%) | 405 (62%) | 109 (17%) | 393 (61%) | 371 (57%) | 649 |
| 8+ | 144 (84%) | 137 (80%) | 70 (41%) | 88 (51%) | 99 (58%) | 115 (67%) | 43 (25%) | 106 (62%) | 114 (66%) | 172 |
| **Total** | 1321 (78%) | 1196 (71%) | 771 (46%) | 855 (50%) | 945 (56%) | 983 (58%) | 291 (17%) | 1034 (61%) | 993 (59%) | 1694 |
| **p-value** | **<0.001** | **<0.001** | **0.02** | **0.428** | **0.658** | **<0.001** | **0.03** | **1** | **0.02** |  |

*p-value from chi-square test unless otherwise noted. **p-value from Cochran-Armitage Trend test.
