# Supplementary material for: Academic information on Twitter: A user survey
Source: PLoS One. 2018 May 17;13(5):e0197265. doi: 10.1371/journal.pone.0197265 (PMC5957360; doi:10.1371/journal.pone.0197265)
Supplement: S3 Table — (DOCX) [file pone.0197265.s003.docx]

# S3 Table. Types of scientific content shared by users on Twitter by discipline, gender, age, and job.

|  | **Research articles and other published works** | **Presentation slides** | **Videos and Images** | **Blog posts** | **Research-related news** | **Lay Summaries** | **Policy announcements** | **Answered question** |
| --- | --- | --- | --- | --- | --- | --- | --- | --- |
| **Academic background** | | | | | | | |  |
| Social Sciences | 495 (80%) | 120 (20%) | 195 (32%) | 423 (69%) | 441 (72%) | 265 (43%) | 220 (36%) | 615 |
| Humanities | 262 (72%) | 62 (17%) | 134 (37%) | 251 (69%) | 221 (60%) | 146 (40%) | 95 (26%) | 366 |
| Engineering/Technology | 174 (73%) | 75 (32%) | 86 (36%) | 155 (65%) | 152 (64%) | 85 (36%) | 55 (23%) | 238 |
| Natural Sciences | 169 (77%) | 37 (17%) | 67 (30%) | 126 (57%) | 158 (72%) | 103 (47%) | 56 (25%) | 220 |
| Medical/Health Sciences | 168 (81%) | 39 (19%) | 59 (28%) | 131 (63%) | 147 (71%) | 94 (45%) | 75 (36%) | 208 |
| Agricultural Sciences | 13 (72%) | 5 (28%) | 7 (39%) | 11 (61%) | 16 (89%) | 10 (56%) | 7 (39%) | 18 |
| **Total** | 1281 (77%) | 338 (20%) | 548 (33%) | 1097 (66%) | 1135 (68%) | 703 (42%) | 508 (31%) | 1665 |
| **p-value** | **0.021** | **<0.001** | **0.267** | **0.082** | **0.001** | **0.099** | **<0.001** |  |
| **Researcher status** | | | | | | | |  |
| Researcher | 780 (82%) | 203 (21%) | 262 (28%) | 597 (63%) | 716 (76%) | 367 (39%) | 281 (30%) | 719 |
| Not researcher | 500 (70%) | 135 (19%) | 286 (40%) | 500 (70%) | 419 (58%) | 336 (47%) | 227 (32%) | 946 |
| **Total** | 1280 (77%) | 338 (20%) | 548 (33%) | 1097 (66%) | 1135 (68%) | 703 (42%) | 508 (31%) | 1665 |
| **p-value** | **<0.001** | **0.284** | **<0.001** | **0.001** | **<0.001** | **<0.001** | **0.239** |  |
| **Year first published manuscript** | | | | | | | |  |
| Median ** | 2007 | 2006 | 2008 | 2007 | 2007 | 2007 | 2006 | 844 |
| (min, max) | (1966, 2016) | (1968, 2016) | (1970, 2016) | (1970, 2016) | (1966, 2016) | (1966, 2016) | (1970, 2016) |  |
| **p-value** | **0.576** | **0.346** | **0.002** | **0.007** | **0.882** | **0.866** | **0.281** |  |
| **Work sector** | | | | | | | |  |
| Academia | 751 (80%) | 193 (21%) | 270 (29%) | 590 (63%) | 707 (75%) | 371 (40%) | 275 (29%) | 939 |
| Government | 47 (73%) | 10 (16%) | 21 (33%) | 44 (69%) | 42 (66%) | 33 (52%) | 23 (36%) | 64 |
| Industry/Professional | 483 (73%) | 135 (20%) | 257 (39%) | 463 (70%) | 386 (58%) | 299 (45%) | 210 (32%) | 662 |
| **Total** | 1281 (77%) | 338 (20%) | 548 (33%) | 1097 (66%) | 1135 (68%) | 703 (42%) | 508 (31%) | 1665 |
| **p-value** | **0.003** | **0.606** | **<0.001** | **0.014** | **<0.001** | **0.028** | **0.397** |  |
| **Current position** | | | | | | | |  |
| Administrator | 116 (66%) | 31 (18%) | 72 (41%) | 117 (66%) | 120 (68%) | 70 (40%) | 59 (34%) | 176 |
| Faculty | 410 (83%) | 98 (20%) | 131 (27%) | 287 (58%) | 378 (77%) | 184 (37%) | 135 (27%) | 493 |
| Journalist | 66 (74%) | 11 (12%) | 33 (37%) | 62 (70%) | 56 (63%) | 46 (52%) | 20 (22%) | 89 |
| Manager | 94 (77%) | 21 (17%) | 45 (37%) | 89 (73%) | 75 (61%) | 53 (43%) | 40 (33%) | 122 |
| Professional | 303 (71%) | 93 (22%) | 163 (38%) | 310 (73%) | 245 (58%) | 207 (49%) | 132 (31%) | 426 |
| Researcher | 166 (83%) | 49 (25%) | 53 (27%) | 133 (67%) | 154 (77%) | 76 (38%) | 68 (34%) | 199 |
| Student | 123 (78%) | 33 (21%) | 50 (32%) | 98 (62%) | 106 (68%) | 65 (41%) | 54 (34%) | 157 |
| **Total** | 1278 (77%) | 336 (20%) | 547 (33%) | 1096 (66%) | 1134 (68%) | 701 (42%) | 508 (31%) | 1662 |
| **p-value** | **<0.001** | **0.243** | **<0.001** | **<0.001** | **<0.001** | **0.011** | **0.208** |  |
| **Gender** | | | | | | | |  |
| Males | 706 (78%) | 200 (22%) | 298 (33%) | 592 (66%) | 591 (66%) | 357 (40%) | 272 (30%) | 900 |
| Females | 566 (75%) | 136 (18%) | 247 (33%) | 500 (66%) | 539 (71%) | 345 (46%) | 232 (31%) | 754 |
| **Total** | 1272 (77%) | 336 (20%) | 545 (33%) | 1092 (66%) | 1130 (68%) | 702 (42%) | 504 (30%) | 1654 |
| **p-value** | **0.095** | **0.034** | **0.865** | **0.873** | **0.013** | **0.012** | **0.824** |  |
| **Age**** | | | | | | | |  |
| <21 | 6 (60%) | 2 (20%) | 6 (60%) | 6 (60%) | 7 (70%) | 3 (30%) | 5 (50%) | 10 |
| 21-30 | 226 (72%) | 58 (18%) | 111 (35%) | 204 (65%) | 199 (63%) | 127 (40%) | 81 (26%) | 314 |
| 31-40 | 421 (80%) | 112 (21%) | 183 (35%) | 362 (69%) | 377 (72%) | 220 (42%) | 151 (29%) | 525 |
| 41-50 | 334 (80%) | 79 (19%) | 130 (31%) | 272 (66%) | 297 (72%) | 166 (40%) | 128 (31%) | 415 |
| 51-60 | 193 (75%) | 55 (21%) | 73 (28%) | 164 (63%) | 160 (62%) | 110 (42%) | 87 (34%) | 259 |
| 60+ | 95 (71%) | 31 (23%) | 42 (31%) | 85 (63%) | 88 (66%) | 72 (54%) | 53 (40%) | 134 |
| **Total** | 1275 (77%) | 337 (20%) | 545 (33%) | 1093 (66%) | 1128 (68%) | 698 (42%) | 505 (30%) | 1657 |
| **p-value** | **0.009** | **0.823** | **0.143** | **0.637** | **0.017** | **0.104** | **0.032** |  |
| **Age of Twitter account, years** | | | | | | | |  |
| <1 | 17 (61%) | 3 (11%) | 9 (32%) | 12 (43%) | 17 (61%) | 9 (32%) | 7 (25%) | 28 |
| 1-2 | 112 (77%) | 23 (16%) | 37 (26%) | 78 (54%) | 102 (70%) | 52 (36%) | 37 (26%) | 145 |
| 2-5 | 530 (77%) | 113 (16%) | 219 (32%) | 437 (64%) | 455 (66%) | 278 (40%) | 219 (32%) | 688 |
| 5-8 | 483 (76%) | 146 (23%) | 214 (34%) | 430 (68%) | 453 (72%) | 294 (47%) | 191 (30%) | 632 |
| 8+ | 136 (81%) | 52 (31%) | 68 (40%) | 138 (82%) | 107 (64%) | 70 (42%) | 54 (32%) | 168 |
| **Total** | 1278 (77%) | 337 (20%) | 547 (33%) | 1095 (66%) | 1134 (68%) | 703 (42%) | 508 (31%) | 1661 |
| **p-value** | **0.216** | **<0.001** | **0.073** | **<0.001** | **0.112** | **0.052** | **0.575** |  |

*P-value from Chi-square test unless otherwise noted. **p-value from Kruskal-Wallis test.
