# Supplementary material for: Academic information on Twitter: A user survey
Source: PLoS One. 2018 May 17;13(5):e0197265. doi: 10.1371/journal.pone.0197265 (PMC5957360; doi:10.1371/journal.pone.0197265)
Supplement: S4 Table — (DOCX) [file pone.0197265.s004.docx]

# S4 Table. The level of scholarly activity on Twitter by discipline, gender, age, and occupation.

| **Characteristic** | **Tweets less than weekly** | **Tweets at least weekly** | **P-value*** |
| --- | --- | --- | --- |
| **N** | 565 (34.1%) | 1092 (65.9%) | - |
| **Academic Background**  Social Sciences  Humanities  Engineering/Technology  Natural Sciences  Medical/Health Sciences  Agricultural Sciences | 1187 (31%)  134 (38%)  91 (38%)  77 (34%)  66 (32%)  10 (56%) | 421 (69%)  223 (62%)  148 (62%)  151 (66%)  141 (68%)  8 (44%) | 0.057 |
| **Self-classified Researcher** | 299 (32%) | 646 (68%) | 0.015 |
| **Years involved in research****  0-1  2-5  5-10  10-20  20+ | 10 (42%)  55 (30%)  84 (32%)  87 (29%)  66 (35%) | 14 (58%)  126 (70%)  177 (68%)  217 (71%)  122 (65%) | 0.117 |
| **Year first article published***, median (min, max)** | 2007 (1964, 2016) | 2006 (1968, 2016) | 0.708 |
| **Sector of work**  Academia  Government  Industry/Professional | 301 (32%)  24 (36%)  239 (37%) | 646 (68%)  42 (64%)  404 (63%) | 0.078 |
| **Current Position**  Administrative Staff  Faculty  Journalist  Manager  Professional Staff  Researcher  Student | 52 (30%)  159 (32%)  17 (20%)  43 (36%)  177 (42%)  56 (28%)  60 (39%) | 121 (70%)  343 (68%)  67 (80%)  76 (64%)  245 (58%)  142 (72%)  95 (61%) | <0.001 |
| **Males** | 290 (32%) | 626 (68%) | 0.020 |
| **Females** | 271(30%) | 459(70%) | 0.020 |
| **Age****  <21  21-30  31-40  41-50  51-60  60+ | 2 (20%)  131 (42%)  175 (33%)  130 (32%)  78 (30%)  45 (33%) | 8 (80%)  181 (58%)  351 (67%)  276 (68%)  179 (70%)  93 (67%) | 0.019 |
| **age of twitter account, years**  <1  1-2  2-5  5-8  8+ | 9 (32%)  59 (41%)  237 (35%)  206 (32%)  50 (29%) | 19 (68%)  85 (59%)  435 (65%)  430 (68%)  121 (71%) | 0.032 |

*p-value from chi-square test unless otherwise noted. **p-value from Cochran-Armitage Trend test. *** Mann-Whitney test.
